# Recurrent bowel-blood translocations of *Escherichia coli* with the unique virulence characteristics over three-year period in the patient with acute myeloid leukaemia – case report

**DOI:** 10.1007/s13353-017-0393-6

**Published:** 2017-03-21

**Authors:** Beata Krawczyk, Anna Śledzińska, Agnieszka Piekarska, Andrzej Hellmann, Józef Kur

**Affiliations:** 1 Department of Molecular Biotechnology and Microbiology, Faculty of Chemistry, Gdańsk University of Technology, Gdańsk, Poland; 2 Department of Therapy Monitoring and Pharmacogenetics, Medical University of Gdansk, Gdańsk, Poland; 3 Department of Hematology and Transplantology, Medical University of Gdańsk, University Clinical Center, Dębinki 7, 80-952 Gdańsk, Poland

**Keywords:** Bacteraemia, Bowel translocation, *Escherichia coli*, Leukaemia, Virulence factors

## Abstract

In patients with haematological malignancies, the bowel remains the main source of *Escherichia coli* bloodstream infections. We present the clinical example of recurrent bowel-blood translocations of *E. coli* with the unique virulence characteristics in a 55-year-old male with the diagnosis of acute myeloid leukaemia. The virulent factors profile of examined strains confirmed that the co-existence of genes *papC*, *sfa*, *usp* and *cnf1*, encoding virulence factors, predisposes *E. coli* to translocation from the gastrointestinal tract to the vascular bed. The close cooperation between haematologists and microbiologists is essential to improve the outcome of patients colonised with highly pathogenic strains.

## Background

Treatment of haematological malignancies with high-dose chemotherapy leads to disruption of the mucosal epithelium and prolonged agranulocytosis. Weakening of the immune barriers makes the patients susceptible to life-threatening bloodstream infections from their own microbiota (Cattaneo et al. 2014; Hamalainen et al. 2008; Olson et al. 2014). The co-occurrence of genes *papC*, *sfa*, *usp* and *cnf1* encoding virulence factors (VFs) could predispose *Escherichia coli* (*E. coli*) to translocation from the gastrointestinal (GI) tract to the vascular bed (Krawczyk et al. 2015). The aim of this study was to investigate whether the unique profile of *E. coli* VFs determines the ability to cross GI-blood barrier and to cause recurrent episodes of bacteraemia. An answer to this question would allow design of specific preventive strategies aiming to reduce patients’ mortality due to *E. coli* bloodstream infections.

## Case report

We present the case of a 55-year-old male, admitted to the Department of Haematology and Transplantology in Gdańsk, with the diagnosis of acute myeloid leukaemia. He was administered the induction chemotherapy according to DAC protocol (Holowiecki et al. 2004). On the 4th day, a high fever with C-reactive protein (CRP) 211 mg/L and procalcitonin (PCT) 8.3 ng/mL was observed. Piperacillin with tazobactam (Tazocin®) treatment was initiated and blood cultures confirmed bacteremia with *E. coli* etiology, sensitive to the administered antibiotic (Culture 1b). After 3 days his temperature normalized. Due to the lack of complete remission (CR), he received a 2nd induction therapy — CLAG-M protocol (Wierzbowska et al. 2008), achieving the first CR (CR1).

He relapsed after 6 months and was given the re-induction therapy according to CLAG-M protocol that resulted in CR2. During the next hospitalization he underwent the first consolidation therapy — HAM protocol (Schlenk et al. 2005). On the 3rd day of agranulocytosis, a high fever appeared with increased CRP (74 mg/L) and PCT (17.3 ng/mL). Tazocin® was initially administered, changed into meropenem after 2 days due to clinical deterioration into septic shock, requiring the pressor therapy. Blood cultures were positive with two isolates: *E. coli* and *S. epidermidis* (Culture 2b). After 2 days he gradually recovered.

He relapsed after 13 months and received re-induction chemotherapy — FLAG-Ida protocol (Hashmi et al. 2005). From the admission day he remained in agranulocytosis and developed febrile infection with CRP 17.9 mg/L and PCT 22.3 ng/mL. Empiric therapy with cefoperazone plus sulbactam (Sulperazon®) was given. Blood cultures were positive with *E. coli* and *E. faecalis* isolates, both sensitive to Sulperazon® (Culture 3b). His clinical status stabilized but his leukaemia appeared to be chemo-resistant and he was disqualified from the intensive treatment.

A few months later the relapse of bladder cancer was diagnosed and transurethral resection of the bladder tumour (TURBT) was performed. After the TURBT procedure he became feverish and cefuroxime was prescribed by the urologists. At that time he was permanently in agranulocytosis. After discontinuation of the antibiotic, a high fever recurred with CRP 250 mg/l and PCT 41.2 ng/mL. Cultures were taken and cefuroxime given again (culture 4b). Despite *E. coli* sensitivity to cefuroxime, there was no clinical response. Therefore, antibiotic therapy was modified to ceftriaxone with amikacin, then changed into Sulperazon®, prescribed and administered at the Daily Haematological Unit. After 6 days of this therapy our patient was admitted to the hospital in critical clinical condition. Because of unfavourable prognosis the treatment was not escalated and he died.

## Material and methods

The collection of rectal swabs and stools was a prospective routine procedure performed in high risk patients once a week (for detection of the drug-resistant bacteria) and in case of the febrile infection. *E. coli* as a part of the physiological microbiome was isolated, identified and cryopreserved from the same material to avoid additional procedures in one patient. The selection of *E. coli* blood isolates for the investigation was based on confirmed episodes of bacteraemia with *E. coli*, with the simultaneous isolation of the microorganism from the stool or urine. *E. coli* isolates were tested by DNA fingerprinting combined with the PCR analysis of VFs. Genotyping of *E. coli* by PCR melting profile and restriction endonuclease analysis using pulsed-field gel electrophoresis were carried out according to Krawczyk et al. (2006). Determination of *E. coli* phylogenetic group was performed with the use of the method by Clermont et al. (2000). PCR screening of virulence genes was based on methodology described by Krawczyk et al. (2015).

## Results

The characteristics of examined strains are presented in Table 1. The genotyping techniques confirmed that *E. coli* isolates from blood shared the same genotype with *E. coli* cultured from the patient’s bowel or urine. We performed the analysis of 21 genes encoding VFs typical for various *E. coli* pathotypes and established their relationship with the phylogenetic group. No genes encoding VFs characteristic for diarrhoeagenic *E. coli* (DEC) were detected in PCR in tested blood isolates. The lack of these VFs was also confirmed in serological tests. Isolates belonged to pathogenic B2 (three episodes) and non-pathogenic group B1 (one episode). The analysis of VFs profiles for examined strains determined the presence of four specific factors previously described, encoded by *papC, sfaD/E, cnf1, usp* genes, and additional three factors, encoded by *agn43, hlyA*, and *iutA* genes, also predisposing to bowel colonization and translocation.

**Table 1:** Molecular characterization of *Escherichia coli* strains isolated from the patient with acute myeloid leukemia during 3-year-period

Sequence of cultures^a^	Source	Genotype PCR MP^b^	Phylogenetic group	Virulence factors^c^													
				*afa/dr*	*fimG/H*	*sfa*	*papC*	*hlyA*	*usp*	*cnf1*	*fyuA*	*iutA*	*ibeA*	*iha*	*focG*	*ksp MTII*	*agn43*
Culture 1s	stool	A	B2	+	+	+	+	−	+	+	+	+	−	−	−	+	+
Culture 1b	blood	A	B2	+	+	+	+	−	+	+	+	+	−	−	−	+	+
Culture 2s	stool	B	B1	+	+	+	+	−	+	+	+	+	+	−	+	−	+
Culture 2b	blood	B	B1	+	+	+	+	−	+	+	+	+	+	−	+	−	+
Culture 3s	stool	C	B2	−	+	+	+	+	+	+	−	+	−	+	−	−	+
Culture 3b	blood	C	B2	−	+	+	+	+	+	+	−	+	−	+	−	−	+
Culture 4b	blood	C	B2	−	+	+	+	+	+	+	−	+	−	+	−	−	+
Culture 4u	urine	C	B2	−	+	+	+	+	+	+	−	+	−	+	−	+	+

^a^s- stool; b- blood; u- urine

^b^PCR melting profile

^c^ *afa/dr* - Dr fimbriae (*afa/draB–C*), *fimG/H* - type 1 fimbriae (*fimG/fimH), sfa* - S fimbriae (*sfaD/sfaE*), *papC* - P fimbriae, *hlyA* - haemolysin, *usp* -bacteriocin Usp, *cnf1* - cytotoxic necrotizing factor, *fyuA* - yersiniabactin receptor, *iutA* - aerobactin receptor, *ibeA* - invasive protein, *iha* - enterobactin (siderofor receptor and adherence factor), *focG* /*H*- F1C fimbriae, *kspMTII* - protein responsible for capsule formation, *agn43* - adhesin 43 (biofilm formation)

To assess the origin of the patient bacterial colonization, the antibiotic resistance profile was performed using sensitive/resistant categories together with MIC values. The results showed sensitivity to the majority of tested antibiotics with the primary resistance to fluoroquinolones (Table 2). Due to a low frequency of multidrug-resistant strains, we concluded that these bacteria were not of hospital origin.

**Table 2:** Analysis of the antibiotic resistance profile among the *E. coli* strains isolated from the patient with acute myeloid leukemia during a 3-year-period

Sequence of cultures	Source	Antibiotic resistance																
		AM	AMC	TZP	CXM	CTX	CAZ	CRO	FEP	SCP	IPM	MEM	AN	GE	NET	CIP	NOR	SXT
Culture 1s	stool	R	I	S	S	S	S	S	S	S	S	S	S	S	S	R	R	R
Culture 1b	blood	R	I	S	S	S	S	S	S	S	S	S	S	S	S	R	R	R
Culture 2s	stool	R	R	S	S	S	S	S	S	S	S	S	S	S	S	R	R	R
Culture 2b	blood	R	R	S	S	S	S	S	S	S	S	S	S	S	S	R	R	R
Culture 3s	stool	R	I	S	S	S	S	S	S	S	S	S	S	S	S	R	R	R
Culture 3b	blood	R	I	S	S	S	S	S	S	S	S	S	S	S	S	R	R	R
Culture 4b	blood	R	I	S	S	S	S	S	S	S	S	S	S	S	S	R	R	R
Culture 4u	urine	R	I	S	S	S	S	S	S	S	S	S	S	S	S	R	R	R

AM: ampicillin; AMC: amoxycillin/clavulan acid; AN: amikacin; CAZ: ceftazidime: CIP: ciprofloxacin; CRO: ceftriaxone; CTX: cefotaxime; CXM: cefuroxime; FEP: cefepime; GE: gentamicin; IPM: imipenem; MEM: meropenem NET: netelmicin; NOR: norfloxacin; SCP: cefoperazone/sulbactam SXT: sulphamethoxazole/trimethoprim; TZP: piperacillin/tazobactam

## Conclusions

The case represents a frequently observed infectious pattern of haematological patients with bacteraemia episodes in the post-chemotherapy period of agranulocytosis. The intestinal microbiota can cause life-threatening septic complications in this specific group of patients (Cattaneo et al. 2014; Olson et al. 2014). The VFs of *E.coli* colonising our patient and the clinical course with bacteraemia sharing the same genotype profile confirmed that the co-existence of genes encoding P fimbriae, S fimbriae, bacteriocin and cytotoxic necrotizing factor seems to predispose *E. coli* to translocation from the GI tract. This clinical example strongly support the previously proposed concept of bowel-blood translocation strains with the unique virulence characteristics (Krawczyk et al. 2015). Since *E.coli* remains the most frequent factor of bloodstream infections in patients with acute leukemia (Cattaneo et al. 2014), the prophylactic use of antibiotics during the agranulocytosis period, necessarily adjusted to the potential virulence and the drug-resistance profile of colonising *E.coli*, would enable to avoid the septic complications. This case justifies the necessity of constant development in the diagnostic field as well as a close cooperation between haematologists and microbiologists to improve the outcome of patients with haematological malignancies.
